# A D-Shaped Photonic Crystal Fiber Refractive Index Sensor Coated with Graphene and Zinc Oxide

**DOI:** 10.3390/s21010071

**Published:** 2020-12-24

**Authors:** Han Liang, Tao Shen, Yue Feng, Hongchen Liu, Wei Han

**Affiliations:** 1 Key Laboratory of Engineering Dielectrics and Its Application, Ministry of Education, Harbin University of Science and Technology, Harbin 150080, China; 1920900054@stu.hrbust.edu.cn (H.L.); YueFeng@hrbust.edu.cn (Y.F.); 2 Heilongjiang Provincial Key Laboratory of Quantum Manipulation & Control, Harbin University of Science and Technology, Harbin 150080, China; 3 Key Laboratory of Intelligent Optical Sensing and Manipulation, Ministry of Education, Nanjing University, Nanjing 210023, China; 4 Digit Fujian Internet-of-Things Laboratory of Environmental Monitoring, Fujian Normal University, Fuzhou 350117, China; charles_han@fjnu.edu.cn; 5 School of Electrical Engineering and Automation, Harbin Institute of Technology, Harbin 150001, China; fenmiao@hit.edu.cn

**Keywords:** photonic crystal fiber, surface plasmon resonance, D-type fiber sensing, graphene, ZnO

## Abstract

A surface plasmon resonance (SPR) sensor based on a D-shaped photonic crystal fiber (PCF) with an uncomplicated structure is proposed to detect the change of refractive index of liquid analytes, and numerical simulation is carried out by the finite element method (FEM). Using silver as the plasmonic metal, the performances of the SPR-PCF sensor coated with a graphene layer and zinc oxide (ZnO) layer were assessed. The sensor designed is only coated with material on the polished surface, which makes the sensor production uncomplicated and solves the problems of filling material in the hole and coating on the hole wall. The effects of structural parameters such as graphene layer thickness, silver layer thickness, ZnO thickness, lattice spacing and manufacturing tolerance of blowhole diameter on the sensor performance were numerically simulated. The numerical results show that the sensitivity of the SPR-PCF sensor coated with 25 nm ZnO is highest in the ZnO thickness range from 10 to 25 nm. In the refractive index range of 1.37–1.41 for liquid analyte, the maximum sensitivity and corresponding resolution reach 6000 nm/RIU and 1.667 × 10^−5^, respectively. In addition, the sensor has good stability and high structural tolerance under the tolerance of ±5% of blowhole diameter. This work has wide application value in the detection of biochemical analytes, water pollution monitoring, food quality, and medical diagnosis.

## 1. Introduction

With the development of high technology, many effective optical fiber sensing technologies for detecting refractive index have been proposed so far, such as fiber grating and fiber interferometer. Surface plasmon resonance (SPR) is widely used because of its high sensitivity [1], high resolution [2], strong selectivity, wide working wavelength range and other [3] advantages, especially in gas detection, food safety [4], biosensor, chemical detection [5,6], environmental monitoring and control, medical [7] and other fields. There are still some problems for all kinds of common optical fiber SPR sensors, such as mode coupling and complex operation. The combination of SPR technology and PCF solves the problems of traditional optical fiber SPR sensor phase matching difficulty [8]. It has the advantages of anti-electromagnetic interference, high sensitivity [9], easy integration and flexible design, as well as has achieved good application results in the fields of nonlinear optics, supercontinuum and sensing [10,11]. Since the first quartz photonic crystal fiber [12] was manufactured in 1996 by Knight and others, great progress has been made in the development theory and manufacturing process. Now, the most widely used mature PCF manufacturing method is the stack stretch method [13].

So far, many scholars have proposed SPR-PCF sensors with simple structures, excellent performance and meeting the actual needs. According to the position of the metal/metal oxide, PCF can be roughly divided into the following categories: first, the metal film is selectively plated around the blowholes of the cladding. Rifat et al. [14] proposed a SPR-PCF sensor with selective filling analyte channel. Its maximum wavelength sensitivity and amplitude sensitivity were 3000 nm/RIU and 418 RIU^−1^, respectively, in the sensing range of 1.46–1.49. Second, metal is selectively filled into the cladding blowholes. Chen et al. [15] proposed a hexagonal SPR-PCF sensor with dual optical flow channels. The central hole is filled with gold, and there are analyte channels on both sides of the gold wire. The results show that when the refractive index of the analyte is 1.32–1.38, the maximum wavelength sensitivity is 5500 nm/RIU, the maximum amplitude sensitivity is 150 RIU^−1^, and the RI resolution is 1.82 × 10^−5^ RIU. The third is to coat the PCF with metal film. Hasan et al. [16] proposed a gold-plated photonic crystal fiber biosensor. Its structure adopted a double layer hole ring, and the missing blowholes are used to produce a double refraction effect. The results show that the maximum sensitivity of the sensor is 2200 nm/RIU in the sensing range of 1.33–1.36 when the thickness of gold layer is 40 nm. Because it is very troublesome to change the physical parameters by injecting or coating metal film into the blowholes in the actual production and processing, some researchers put forward another method to coat the metal film on the polishing plane of PCF. Tong et al. [17] proposed a highly sensitive D-type SPR-PCF biosensor. Two small blowholes in the center facilitate the phase matching between the plasma exciton and the fiber core. In the refractive index range of 1.34–1.40, the average sensitivity of the sensor is 4850 nm/RIU, and the resolution is as high as 2 × 10^−5^ RIU.

In actual sensing field applications, such as biomedicine, food safety or environmental chemistry, the equipment needs to be very sensitive to the RI changes of some unknown analytes. Therefore, this paper proposes and analyzes a highly sensitive D-type PCF-SPR refractive index sensor with a double-layer composite film. The sensor can work in the near infrared region. Taking the chemical stability of the excitation material and the resonance output energy into account, this paper adopts the method of coating a composite film on the outside of the PCF, depositing a layer of silver on the polished surface of the PCF as the plasmonic exciton source material, and then coating graphene or zinc oxide to prevent metal film from being oxidized. In order to obtain a larger detection range and higher sensitivity, we discuss the performance of PCFs of these two material combinations (silver-graphene and silver-ZnO). The simulation results show that the performance of the sensor with ZnO coating is better than that with graphene coating. The finite element analysis method is used to carry out a numerical analysis of blowhole size, lattice spacing, material thickness, etc., to discuss the sensitivity of the refractive index changes of the analyte.

## 2. Model Structure and Production Potential

**Figure 1:**
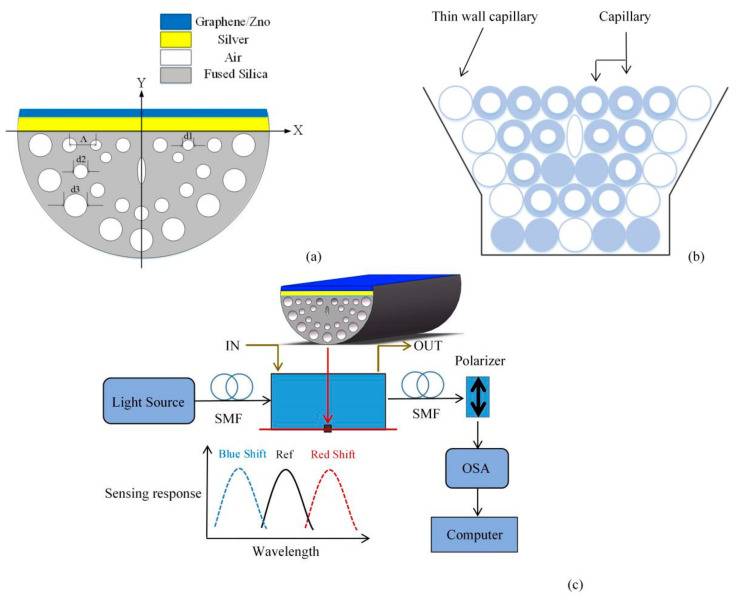
(**a**) The cross-section of the sensor; (**b**) stacked rough machining cross-section; (**c**) experimental setup of SPR sensor for detecting the refractive index of liquid analytes.

Based on the the full-vectorial FEM, the two-dimensional waveguide section of the PCF is drawn in the electromagnetic wave, frequency domain physics field of the wave optics module of COMSOL Multiphysics software, and more accurate mode field analysis results are obtained through finer grid calculation. The two-dimensional cross-section, stack simulation cross-section and experimental device of the sensor are shown in Figure 1a–c. The whole structure can be simply divided into two parts. The first part is composed of blowholes and fused quartz which is used as background material. The pores are divided into three layers, formed by the other six pores in the same row as the two pores above the core, which are respectively rotated by ±20°, ±40°, ±60° with the coordinate origin as the center. The diameter of PCF is 10 µm. The size of the four holes in the first layer is the same, *d*_1_ = 0.5 µm. The elliptical pores at the core and the first layer of pores disappearing on both sides cause a strong birefringence effect, which makes the confinement loss and sensitivity of *y*-polarized light far stronger than *x*-polarized light. The two pores above the core and the other six pores in the same row are used to adjust the intensity of the evanescent wave that interacts with the surface plasmon polariton (SPP) mode. The blowholes in the second layer and the third layer are larger, with diameters of *d*_2_ = 0.7 µm and *d*_3_ = 1 µm, respectively, which are used to limit the propagation of light. The lattice spacing is *Λ* = 2 µm. The second part is SPR excitation layer. Ag is used as SPR excitation material, and its thickness is 30 nm. In order to enhance the sensing performance and prevent silver oxidation, graphene or zinc oxide is coated to form a double-layer composite film. The thickness of graphene is *L* × 0.34 nm, where *L* is the number of graphene layers. The thickness of ZnO layer is expressed as *tz* = 20 nm. The phase matching position changes with the modification of the analyte refractive index. The constant analyte thickness is 1.0 µm. In order to achieve higher accuracy, scattering boundary conditions are added to prevent the reflection of light from interfering with the fiber mode. Triangular mesh and boundary layer mesh are used to divide the calculation area, and the degree of freedom is 168,591.

The background material of PCF is fused quartz, and the definition of its refractive index is given by Sellmeier [18] equation:

(1) $$n^{2}(\lambda )=1+\frac{A_{1}\lambda^{2}}{\lambda^{2}-C_{1}}+\frac{A_{2}\lambda^{2}}{\lambda^{2}-C_{2}}+\frac{A_{3}\lambda^{2}}{\lambda^{2}-C_{3}}$$

where *A*_1_ = 0.69616300, *A*_2_ = 0.407942600, *A*_3_ = 0.897479400, *C*_1_ = 0.00467914826, *C*_2_ = 0.0135120631, *C*_3_ = 97.9340025. Graphene coating can improve sensing performance due to its high surface to volume ratio, broadband optical and plasmonic characteristics [19,20]. In addition, it can increase the absorption of analyte molecules by stacking, with good plasma properties, which are suitable for sensing. The refractive index of graphene is obtained as follows [21]:

(2) $$n_{g}=3+iB_{1}\lambda /3$$

where, *B*_1_ ≈ 5.446 µm^−1^. In addition, the Drude dispersion model is used to describe the dielectric constant of Ag [22]:

(3) $$\varepsilon_{Ag}=\varepsilon_{\gamma }+i\varepsilon_{i}=1-\frac{\lambda^{2}\lambda_{c}}{\lambda_{p}^{2}(\lambda_{c}+i\lambda )}$$

where, *λ_p_* = 0.14541, *λ_c_* = 17.6140. ZnO is one of the most important functional oxides because of its high binding energy [23,24] and stable chemical properties in the interaction with liquid analytes. Therefore, using an ultra-thin ZnO layer with optimized thickness on the metal layer can improve the sensitivity of the sensor, detect specific analytes, and protect the metal layer from oxidation [25]. The dielectric constant of ZnO can be obtained by the following formula [26]:

(4) $$\varepsilon_{ZnO}=\varepsilon_{r}+i\varepsilon_{i}=2.81418-\frac{B_{1}\lambda^{2}}{(\lambda^{2}-B_{2}^{2})-B_{3}^{2}}$$

where, *B*_1_ = 0.87968, *B*_2_ = 0.3042, *B*_3_ = 0.00711. Confinement loss [27] is a key parameter to evaluate the performance of the sensor. It is directly proportional to the imaginary part Im(*n_eff_*) of the effective refractive index of the mode, and is defined as:

(5) αloss=40×π×Im(neff)×104λ×ln(10)(dB/cm)

According to the loss spectrum, confirm the maximum loss value to determine the corresponding resonance wavelength. By analyzing the change of the loss spectrum caused by the change of the refractive index of the medium, the perception and detection of the medium state can be accurately realized.

It should be noted that the sensitivity of the sensor is measured by wavelength detection in this work.

The sensitivity of wavelength detection [28] can be calculated by Equation (6):

(6) $$S_{\lambda }=\frac{\Delta \lambda_{peak}}{\Delta n_{a}}(\mathrm{nm}/\mathrm{RIU})$$

Δ*λ_peak_* represents the change of resonance wavelength and Δ*n_a_* represents the change of refractive index of the object to be measured. Wavelength resolution is another important parameter that determines the detection degree of RI change of analyte, which can be determined by the following equation [29]:

(7) $$R=\frac{\Delta n_{a}\times \Delta \lambda_{\min}}{\Delta \lambda_{peak}}(\mathrm{RIU})$$

where Δ*λ_min_* represents the wavelength resolution and can be set to 0.1 nm.

It is feasible to manufacture the proposed PCF by the standard stack-stretch method. Firstly, the quartz sleeve is pretreated. The capillaries are manufactured according to the parameters in an ultra-clean environment and then both ends of the capillary are tapered with a hydrogen-oxygen flame to seal the holes. The capillaries are stacked in the quartz sleeve according to the design requirements to form the required structure, and the gaps are filled with pure quartz rods. The quartz sleeve and the capillary tube are sintered together with an oxyacetylene flame, and the PCF is made by wire drawing technology on the drawing tower. Finally, part of the cladding layer on the side of the PCF is smoothly polished by etching or side polishing, as shown in Figure 1b. In terms of polishing technology, wheel polishing method and V-groove polishing method have become more and more mature [30]. Metal layer can be deposited on the polished surface of PCF by chemical vapor deposition, high pressure microfluidic chemical deposition and sputtering methods [31,32,33]. Kiraly et al. [34] reported that single-layer graphene was successfully grown on single crystal Ag (111) by evaporating atomic carbon under ultra-high pressure. Kim et al. [35] reported a method of direct synthesis of large-scale graphene thin films, and proposed two different methods to prepare the films and transfer them to any substrate. The quality of graphene grown by chemical vapor deposition is as high as that of mechanically cut graphene, and the number of graphene layers can be controlled. Tiwale et al. [36] reported a method for preparing ZnO thin films from new zinc decanoate. Uniform zinc oxide coatings can also be obtained by DC magnetron sputtering, thermal evaporation and RF magnetron sputtering [25,37,38]. The schematic diagram of RI sensing systerm is described in Figure 1c. A wide-band light source can be used to launch light into the single mode fiber (SMF) and couple with the D-type PCF. The analyte flow channel facilitates the in and out of analytes. Then connect the D-type PCF with the polarizer through a SMF to transmit the *Y*-polarized light to an optical spectrum analyzer (OSA). The transmission spectra are acquired and analyzed by the OSA and computer.

## 3. Results Analysis and Discussion

### 3.1. Mode Coupling

**Figure 2:**
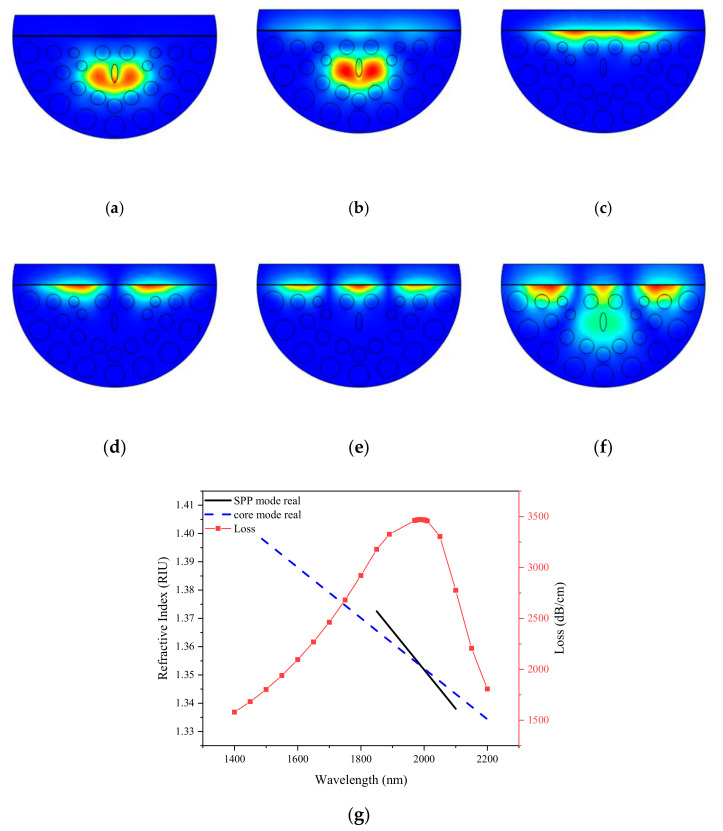
The simulated (**a**) *X*-polarized core mode; (**b**) *Y*-polarized core mode; (**c**) 1-SPP mode; (**d**) 2-SPP mode; (**e**) 3-SPP mode; (**f**) Coupling mode; (**g**) The real part (dispersion) of the effective refractive index under the core mode and SPP mode, and the change of the loss along the y-polarization direction with the wavelength. (*d*_1_ = 0.5 µm, *d*_2_ = 0.7 µm, *d*_3_ = 1 µm, *Λ* = 2 µm, *tz* = 25 nm, RI = 1.40, *ts* = 30 nm).

Figure 2a,b show the simulated *X*-polarized core mode and *Y*-polarized core mode, respectively, where the light is restricted to the core area. The coupling efficiency between the SPP mode and the core mode in *Y* polarization is stronger than in *X* polarization. Therefore, we consider *Y*-polarized core mode for study. There exists a first-order SPP mode, a second-order SPP mode, and a third-order SPP mode in the PCF-SPR sensor proposed as shown in Figure 2c (1-SPP mode), Figure 2d (2-SPP mode) and Figure 2e (3-SPP) mode. The 3-SPP mode is utilized to generate sensor signals in this work. Figure 2f depicts the coupled mode. At this time, the core mode is phase-matched with the SPP mode, a resonance peak will appear, and the energy in the core mode will be transferred to the SPP mode. Figure 2g shows the real part (dispersion) of the effective refractive index under the core mode and SPP mode, and the change of the loss along the y-polarization direction with the wavelength. The blue line indicates the real part of the effective refractive index of the fundamental mode. The black line represents the real part of the effective index of SPP mode. The red curve represents the confinement loss of the core mode. As the energy is transferred from the fundamental mode to the SPP mode, the loss increases with the increase of wavelength. When the wavelength is 1990 nm, the peak loss reaches 3470.63 dB/cm. When the wavelength is greater than 1990 nm, the energy is transferred from SPP mode to fundamental mode, and the loss presents a decreasing trend. The dispersion (the real part of the effective refractive index) of the fundamental mode and SPP mode also intersects at the position of 1990 nm, indicating that the two modes are coupled. The change of analyte refractive index is detected by measuring the drift value of resonance wavelength.

### 3.2. Numerical Analysis of Silver-Graphene PCF

**Figure 3:**
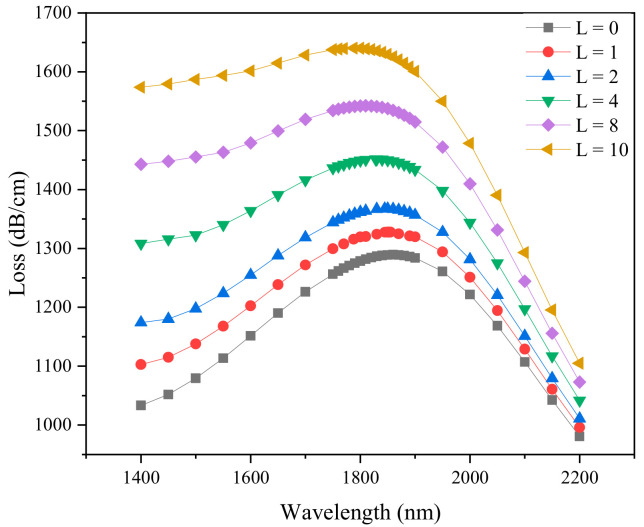
Loss spectra obtained by simulation when the number of layers of graphene (*L*) is 0, 1, 2, 4, 8 and 10. (*d*_1_ = 0.5 µm, *d*_2_ = 0.7 µm, *d*_3_ = 1 µm, *Λ* = 2 µm, *ts* = 30 nm, RI = 1.38).

The thickness of the material layer may affect SPP mode, thereby changing the performance of the sensor. The loss spectrum due to the change in the number of graphene layers is shown in Figure 3 with the parameters of *d*_1_ = 0.5 µm, *d*_2_ = 0.7 µm, *d*_3_ = 1 µm, RI = 1.38 and *ts* = 30 nm. With the increase of the thickness of graphene layer, the resonance peak shifts to shorter wavelength, the wavelength variation Δ*λ*_0–10_ = 72 nm, and the loss change is Δ*α*_0–10_ = 350.88 dB/cm. The thicker the graphene, the more energy coupled from the core mode to the SPP mode, however, also the wider the loss resonance curve. With the thickness of graphene increases by a certain amount, its properties gradually become graphite. In a certain wavelength range, the coupling efficiency between the core mode and the SPP mode is reduced.

**Figure 4:**
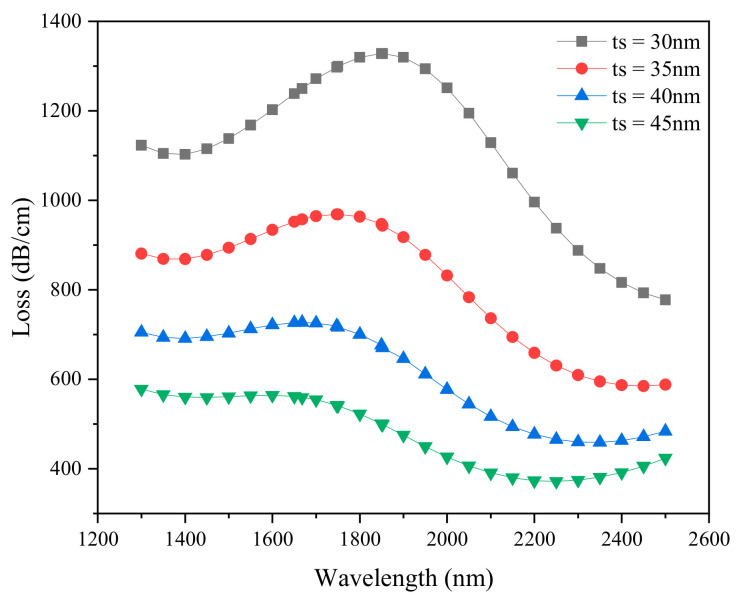
Loss spectrum of silver layer thickness which changes with wavelength obtained by simulation. (*d*_1_ = 0.5 µm, *d*_2_ = 0.7 µm, *d*_3_ = 1 µm, *Λ* = 2 µm, *L* = 1, RI = 1.38).

Although the sensor without graphene layer has high sensitivity, the oxidation of silver makes it difficult to use effectively. The thickness of the plasmonic material has a potential impact on sensor sensitivity. With other parameters kept unchanged, the loss spectrum of the silver layer thickness from 30 to 45 nm was studied, as shown in Figure 4. It can be seen that when the thickness of the silver layer (*ts*) increases from 30 to 45 nm, the resonance wavelength tends to the short wavelength direction, and changes from 1852 to 1600 nm, also the loss peak decreases from 1327.71 to 564.25 dB/cm. When the silver film is too thick, there is more damping loss, which weakens the SPR effect and finally reduces the loss peak. The thickness of silver layer is fixed to 30 nm to discuss the effect of analyte RI on the performance of the sensor.

**Figure 5:**
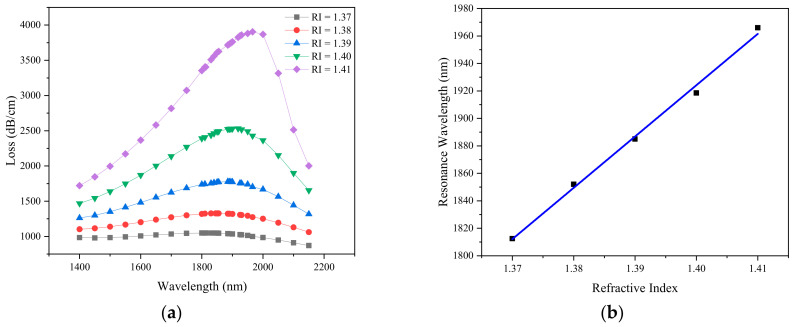
(**a**) Loss spectra obtained by simulation when analyte RI is 1.37–1.41; (**b**) Linear fitting between resonance wavelength and analyte RI. (*d*_1_ = 0.5 µm, *d*_2_ = 0.7 µm, *d*_3_ = 1 µm, *Λ* = 2 µm, *ts* = 30 nm, *L* = 1).

Due to the small change of the analyte RI, the real part of the effective refractive index of the SPP mode changes, which leads to the change of phase matching wavelength between the core mode and SPP mode. The liquid analyte RI range from 1.37 to 1.41 is simulated to investigate the sensing performance of the PCF sensor. Figure 5a depicts the variation of the loss spectra as the analyte RI is changed from 1.37 to 1.41 with *d*_1_ = 0.5 µm, *d*_2_ = 0.7 µm, *d*_3_ = 1 µm, *Λ* = 2 µm, *ts* = 30 nm, *L* = 1. With the increase of refractive index of liquid analyte, the resonance wavelength shifts from 1812.5 to 1966 nm, and the interaction between metal and analyte increases. Higher energy exchange occurs from the fundamental mode to the SPP mode, which also means stronger sensitivity. The wavelength shift is 153.5 nm and the loss shift is 2854.87 dB/cm. Figure 5b shows the resonance wavelength as a function of the refractive index of the liquid analyte. It is found that the maximum wavelength sensitivity of sliver-graphene PCF is 4750 nm/RIU and the maximum wavelength resolution is 2.105 × 10^−5^ RIU when the analyte RI changes from 1.40 to 1.41. The average sensitivity of the sensor is 3735 nm/RIU, and the goodness of fit parameter R^2^ is 0.995, which provides the ability to accurately detect analytes. The fitting equation can be expressed as follows: *y* = 3735*x* − 3304. Maximum confinement loss for different analyte Ris and average sensitivity of the sensor are shown in Table 1.

### 3.3. Numerical Analysis of Silver-ZnO PCF

**Figure 6:**
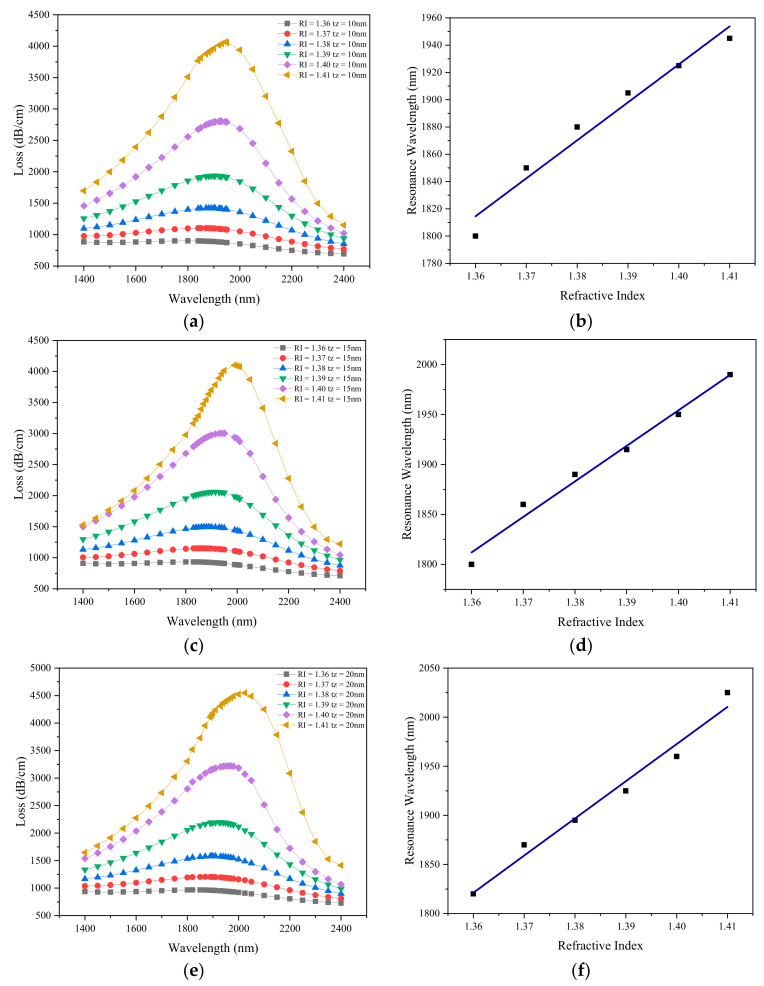

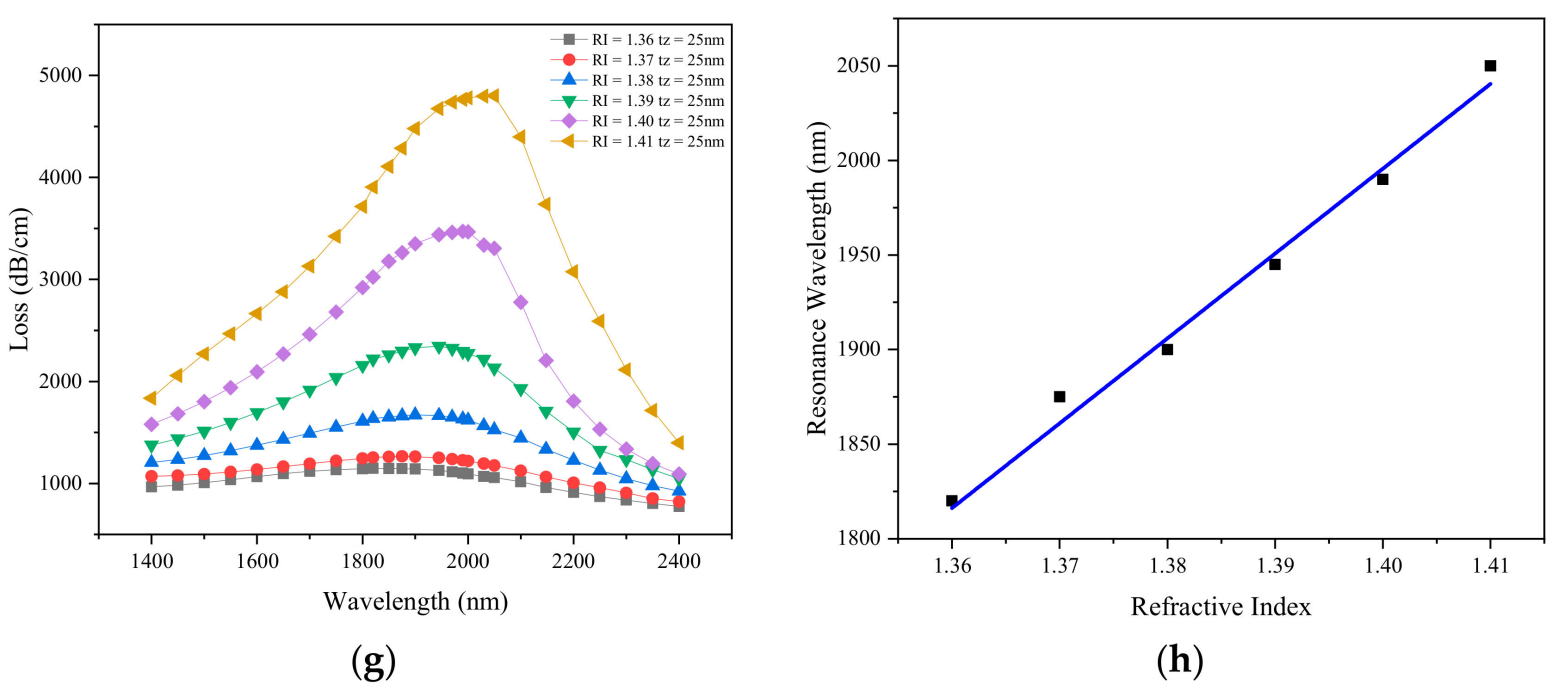
Loss spectra obtained by simulation at analyte RI of 1.37–1.41 when zinc oxide thickness is (**a**) 10 nm; (**b**) 15 nm; (**c**) 20 nm; (**d**) 25 nm; Linear fitting of resonance wavelength with analyte RI from 1.37 to 1.41 by simulation when zinc oxide thickness is (**e**) 10 nm; (**f**) 15 nm; (**g**) 20 nm; (**h**) 25 nm. (*d*_1_ = 0.5 µm, *d*_2_ = 0.7 µm, *d*_3_ = 1 µm, *Λ* = 2 µm, *ts* = 30 nm).

Next, we will change graphene into ZnO to be deposited on the silver layer to investigate the influence of ZnO thickness on sensor’s performance. The loss spectrum changes with wavelength when the thickness of zinc oxide is 10, 15, 20 and 25 nm is shown in Figure 6a–d. It can be seen that with the increase of the analyte RI, the resonance spectrum shifts to a longer wavelength direction, and the amplitude of the loss spectrum increases gradually. This is because that the increase of the analyte RI reduces the refractive index contrast between the core and the SPP mode [39], and enhances the evanescent field, resulting in stronger coupling. At the same time, larger analyte RI can increase the real part of the effective refractive index of the SPP mode, while that of the core mode remains unchanged, resulting in the phase matching point moving to a longer wavelength. Figure 6e–h illustrates linear fitting of resonant wavelength to analyte RI under different thickness of zinc oxide. The slope of the fitting equation is the refractive index sensitivity. When the thickness of ZnO is *tz* = 10 nm, as shown from Figure 6e, the fitting equation is *y* = 2875.7*x* − 1974. When the thickness of ZnO is *tz* = 15 nm, as shown from Figure 6f, the fitting equation is *y* = 3557.1*x*-3025. When the thickness of ZnO is *tz* = 20 nm, as shown from Figure 6g, the fitting equation is *y* = 3785.7*x* − 3327. When the thickness of ZnO is *tz* = 25 nm, as shown from Figure 6h, the fitting equation is *y* = 4485.7*x* − 4284.

It is found that with the increase of the thickness of ZnO layer, the average sensitivity of the sensor gradually increases. Since ZnO has a large dielectric constant and real part of the refractive index, as the thickness of the ZnO film increases, the confinement loss peak becomes a higher value. The change in resonance wavelength is Δ*λ_peak_* = 60 nm, when the thickness of zinc oxide *tz* = 25 nm and the RI of the analyte ranges from 1.40 to 1.41. According to Equations (6) and (7), the wavelength sensitivity and resolution are 6000 nm/RIU and 1.667 × 10^−5^ RIU. The R^2^ of the fitting equation is greater than 0.99, indicating that the linearity is high. This requires the precision of coating process to control the thickness and ensure the flatness of the film. Table 2 shows the maximum confinement loss and average sensitivity as a function of silver layer thickness and liquid analyte RI.

ComparingFigure 5b and Figure 6h, under the same structure parameters and the same liquid environment, the sensitivity of the sensor coated with graphene is lower than that of the sensor coated with ZnO.

**Figure 7:**
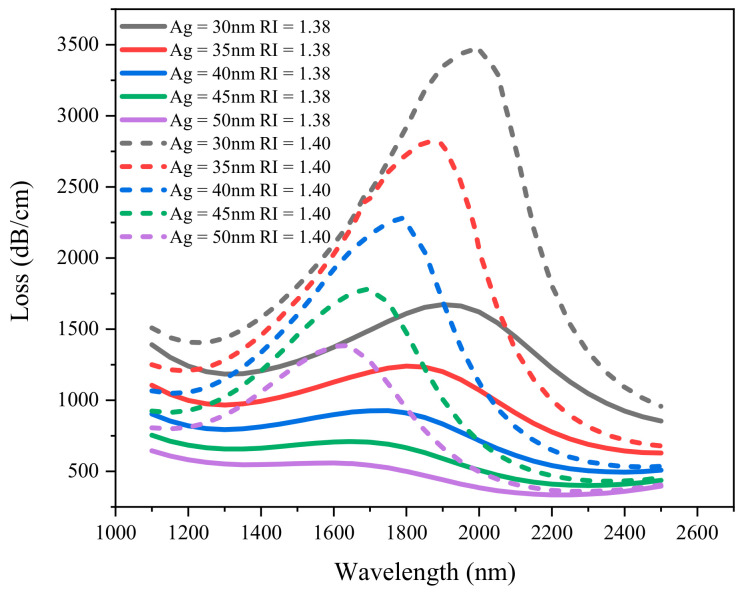
Loss spectrum of silver layer thickness obtained by simulation when the refractive index of liquid is 1.38 and 1.40. (*d*_1_ = 0.5 µm, *d*_2_ = 0.7 µm, *d*_3_ = 1 µm, *Λ* = 2 µm, *tz* = 25 nm).

Based on the above investigation, the optimized ZnO thickness is 25 nm. As an important factor of SPR, the thickness of the deposited silver layer determines the final sensing performance of the sensor. Figure 7 depicts the loss spectrum of the silver layer thickness *ts* = 30–50 nm when the refractive index of liquid analyte is 1.38 and 1.40. It can be clearly seen that with the increase of RI, the loss peak wavelength moves to the long wavelength direction, and the intensity of resonance peak increases gradually. When the thickness of silver layer is 30, 35, 40, 45 and 50 nm, the corresponding wavelength changes are 90, 50, 45, 40 and 40 nm, and the loss peak values are 1798.59, 1578.27, 1355.47, 1069.42 and 825.40 dB/cm. When the analyte RI is fixed, with the increase of the thickness of the silver layer, the resonance wavelength and the intensity of the resonance peak gradually decrease. This is because that with the increase of film thickness, the light penetration through the coating decreases, and the coupling energy between the fundamental mode and SPP mode is lower. If the thickness of the silver layer is too small, part of the energy will pass through the metal layer. Therefore, the optimized silver layer thickness *ts* = 30 nm is selected.

**Figure 8:**
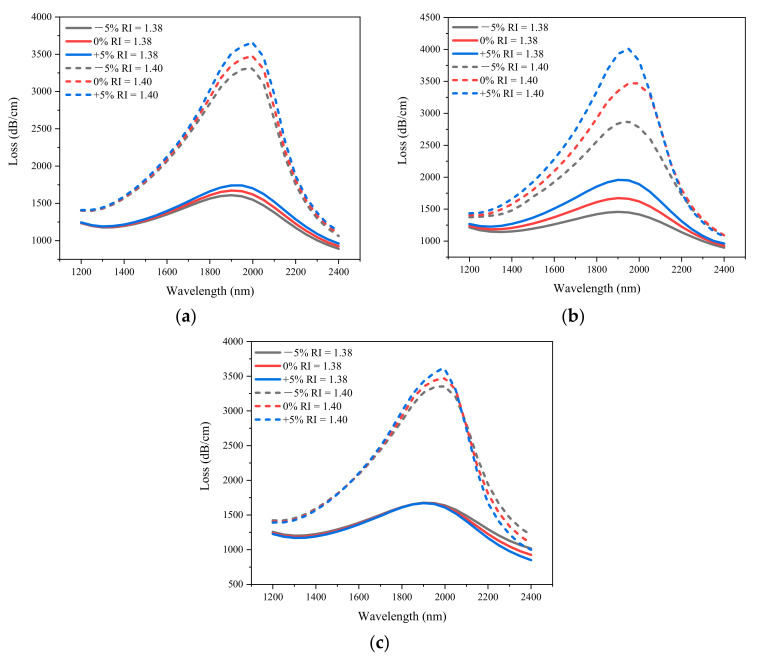
The effect of diameter on confinement loss obtained by simulation. (**a**) *d*_1_; (**b**) *d*_2_; (**c**) *d*_3_. (The solid line represents the confinement loss when the analyte RI is 1.38, and the dotted line represents the confinement loss when the analyte RI is 1.40; *Λ* = 2 µm, *tz* = 25 nm, *ts* = 30 nm.).

The change of blowhole diameter has great influence on the performance of the PCF-SPR sensor. However, in the actual manufacturing process, there is a certain manufacturing tolerance for the blowhole diameter. In order to analyze the influence of structural deformation on the sensor performance and optimize the maximum sensing performance, the ±5% variation of design parameters is considered in this paper. Figure 8 shows the effect of blowhole variation on the sensor performance at RI of 1.38 and 1.40. The solid line represents the confinement loss when the analyte RI is 1.38, and the dotted line represents the confinement loss when the analyte RI is 1.40. With the increase of liquid RI, the resonance wavelength always shifts to the long wavelength at all values of diameter, and the resonance loss increases with the increase of diameter, which enhances the interaction between core mode and SPP mode. Figure 8a shows the effect of *d*_1_ on confinement loss. When *d*_1_ changes by ±5%, the resonant wavelength does not change significantly, and the resonant loss will shift a little. When RI is 1.38 and 1.40, the changes are ±80 dB/cm and ±160 dB/cm, respectively. Figure 8b shows the effect of *d*_2_ on confinement loss. The resonance loss increases with the increase of diameter *d*_2_, that is, the resonance intensity increases with the increase of blowhole size. In actual manufacturing, adjust the loss peak value by changing *d*_2_. The resonance wavelength does not shift with the increase of the hole, which means that the wavelength sensitivity has a very high tolerance to the size of the hole. Figure 8c shows the effect of *d*_3_ on confinement loss. When *d*_3_ changes by ±5%, there is no obvious change in resonance loss and resonance wavelength. In this way, without affecting the sensitivity, the resonant loss intensity suitable for signal detection can be obtained by adjusting the blowhole size, which will improve the detection accuracy to a certain extent.

The work aims to propose a PCF sensor with simple structure, convenient preparation and practical processing. Although the sensitivity of the existing research is better than that proposed in this paper, as shown in Table 3, there are still problems with narrow detection range, complex production techniques of hole wall coating and hole filling with analyte, and higher cost, for example, unpolished PCF requires more material than polished PCF.

Li et al. reported a H-shaped PCF [1] coated with Ag-graphene layers. The open U-shaped groove structure simplifies the preparation process of the material layer. Tong et al. proposed a D-shaped PCF [17] based on silver-graphene in which graphene is added between the silver layer and the PCF to allow the analyte to directly contact the silver. A plasmonic sensor based on a dual-side polished PCF is proposed by Chen et al. [27]. When the refractive index of the analyte changes from 1.395 to 1.415, an RI resolution of 9.39 × 10^−6^ and an average wavelength sensitivity of 10,650 nm/RIU are achieved. Liu et al. reported on symmetrical dual D-shaped PCF [28], where the plasmonic material is placed on a vertical plane. The results show that, compared with a single PCF, the directional power coupling between two PCFs significantly improves the performance of the sensor. Haider et al. [39] proposed a highly amplitude-sensitive PCF sensor. The proposed sensor exhibits the maximum wavelength sensitivity of 18,000 nm/RIU with the sensor resolution of 5.6 × 10^−6^ RIU. Dash et al. reported a similar study [40]. It is worth noting that they let graphene come into contact with silver, and the results show that the sensor without graphene is more sensitive. Yang et al. reported the D-shaped MOF biosensor [41] to realize the simultaneous measurement of refractive index and temperature. Liu et al. proposed a D-type PCF [42] based on a circular layout, which has good sensitivity and preparation performance, and can be extensively utilized in the field of environmental sensing. Shukla et al. proposed a double-layer metal-zinc oxide optical fiber sensor [43]. The results show that the top layer of zinc oxide protects the metal layer from oxidation and improves the sensitivity of the SPR sensor. Momota et al. proposed a hollow-core silver coated PCF [44]. The plasmonic material and sensing layer are placed outside the PCF surface, which facilitates manufacturing and analyte detection. Peng et al. reported a gold-plated film with a rectangular lattice D-type microstructure fiber [45], which has a narrower spectral width and a higher FOM. Islam et al. [46] proposed a dual-polarized highly sensitive plasmonic sensor in the visible to near-IR spectrum. The simulation results show that the maximum wavelength sensitivity of y polarization is 62,000 nm/RIU. This is the highest sensitivity of SPR in the published literature. A simple multi-core flat fiber SPR sensor for refractive index detection suitable for telecommunication wavelengths was proposed by Rifat et al. [47]. In the sensing range of 1.46–1.485, the average wavelength interrogation sensitivity and the maximum sensitivity are 9600 nm/RIU and 23,000 nm/RIU, respectively. Wu et al. [48] proposed a SPR biosensor based on gold-coated side-polished hexagonal structure PCF. The sensitivity is obtained to be as high as 21,700 nm/RIU in the refractive index environment of 1.33–1.34. An et al. [49] reported a D-shaped PCF refractive index sensor based on SPR. The maximum sensitivity obtained is 10,493 nm/RIU with a very high resolution of 9.53 × 10^−6^ RIU in the detection range of 1.33–1.38. Xie et al. [50] investigated the sensing characteristics of the novel D-type SPR-PCF sensor with different side-polished lengths. In the environment with a refractive index of 1.40–1.42, the highest sensitivity can reach 7381.0 nm/RIU.

## 4. Conclusions

The full vector finite element method (FEM) is used for the numerical simulation of a silver-graphene and a silver-ZnO PCF-SPR refractive index sensor. Dielectric materials (graphene/ZnO) are used to inhibit the oxidation of active plasma material (silver). The open structure of side polishing reduces the manufacturing complexity. The simulation results show that the maximum sensitivity of the sensor is 6000 nm/RIU and the resolution is 1.667 × 10^−5^ RIU. It has good stability in the range of ±5% variation of blowhole size. Compared with other types of PCF-SPR sensors, the D-shaped sensor proposed here has the advantages of simple manufacturing, high sensitivity, low cost and reusability, which can be widely used in sample detection of such as life science research, biochemistry, environment and other fields.

## Figures and Tables

**Table 1:** Maximum confinement loss for different analyte RIs and average sensitivity of the sensor.

The Number of Layers of Graphene	Confinement Loss (dB/cm)					Average Sensitivity (nm/RIU)
	RI = 1.37	RI = 1.38	RI = 1.39	RI = 1.40	RI = 1.41	
*L* = 1	1049.03	1327.71	1776.89	2531.53	3903.90	3735

**Table 2:** Maximum confinement loss of the sensor for different thickness of zinc oxide and analyte RIs and corresponding average sensitivity.

ZnO Layer Thickness	Confinement Loss (dB/cm)						Average Sensitivity (nm/RIU)
	RI = 1.36	RI = 1.37	RI = 1.38	RI = 1.39	RI = 1.40	RI = 1.41	
*tz* = 10 nm	901.09	1103.63	1421.60	1936.75	2803.56	4066.57	2785.7
*tz* = 15 nm	933.04	1152.23	1497.43	2059.81	3004.35	4101.73	3557.1
*tz* = 20 nm	968.25	1205.35	1581.01	2195.58	3222.86	4548.06	3785.7
*tz* = 25 nm	1149.43	1265.29	1672.04	2345.52	3470.62	44,738.72	4485.7

**Table 3:** Performance comparison of fiber optic sensors based on SPR.

Feature	RI Range (RIU)	Average Sensitivity (nm/RIU)	Resolution (RIU)	Ref.
H-shaped PCF coated with silver and graphene	1.33–1.36	2770	3.61 × 10^−6^	[1]
D-shaped PCF based on silver-graphene	1.34–1.40	4850	2 × 10^−5^	[17]
Dual-side polished PCF	1.395–1.415	10,650	9.39 × 10^−6^	[27]
Symmetrical dual D-shape PCF	1.36–1.41	14,660	6.82 × 10^−5^	[28]
SPR-PCF based on Au	1.33–1.41	18,000 (max)	5.6 × 10^−6^	[39]
Silver-graphene coated D-shaped PCF	1.33–1.37	3700	2.7 × 10^−5^	[40]
D-shaped MOF biosensor	1.33–1.36	2214	4.51 × 10^−5^	[41]
Au coated D-shaped PCF	1.32–1.35	4000	3.31 × 10^−5^	[42]
Au/Ag/Au-ZnO D-shaped PCF	1.30–1.37	3161 3054 3018	-	[43]
Hollow-core silver coated PCF	1.33–1.37	4200 (max)	2.38 × 10^−6^	[44]
Rectangular lattice D-shaped fiber	1.37–1.395	4500	2.2 × 10^−5^	[45]
Dual-polarized highly sensitive plasmonic sensor	1.33–1.43	62,000 (max)	1.6 × 10^−6^	[46]
Au-TiO_2_ multi-core flat fiber SPR sensor	1.46–1.485	9600	1.22 × 10^−5^	[47]
hexagonal structure PCF based on gold-coated side-polished	1.33–1.34	21,700 (max)	-	[48]
D-shaped PCF based on Au	1.33–1.38	10,493 (max)	9.53 × 10^−6^	[49]
A novel D-type SPR-PCF sensor with different side-polished lengths	1.40–1.42	7381.0 (max)	-	[50]
Our proposed Ag-graphene coated D-PCF	1.37–1.41	3735	2.105 × 10^−5^	-
Our proposed Ag-ZnO coated D-PCF	1.36–1.41	4485.7	1.667 × 10^−5^	-

## Data Availability

No new data were created or analyzed in this study. Data sharing is not applicable to this article.
